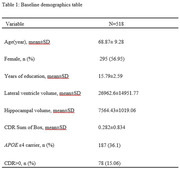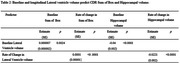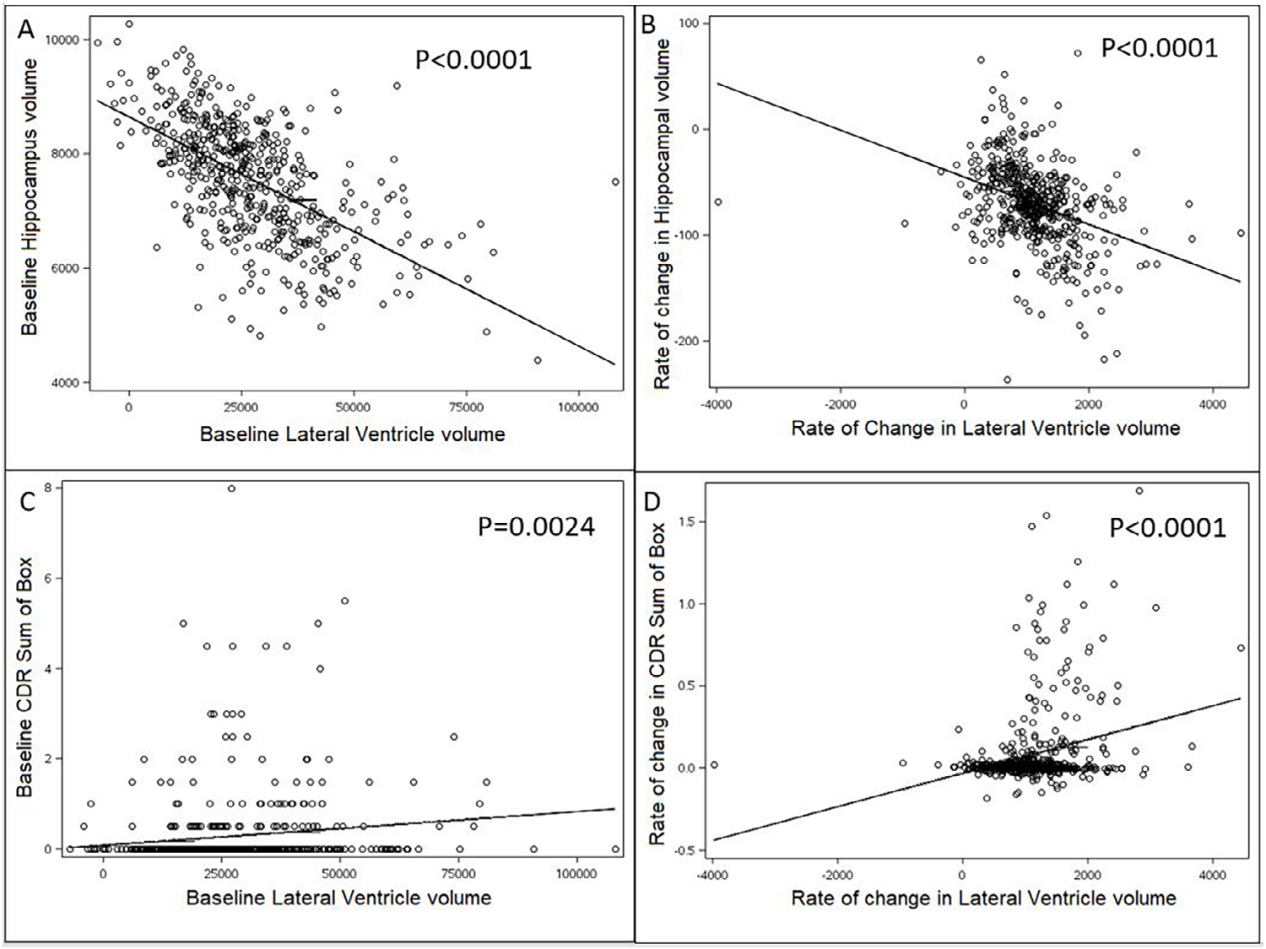# Lateral Ventricle Volumes Predict Hippocampal Volume and Cognition

**DOI:** 10.1002/alz.090469

**Published:** 2025-01-09

**Authors:** Gengsheng Chen, Stephanie Doering, Nelly Joseph‐Mathurin, Parinaz Massoumzadeh, Jingxia Liu, Qing Wang, Brian A. Gordon, John C. Morris, Tammie L.S. Benzinger

**Affiliations:** ^1^ Washington University in St. Louis, School of Medicine, St. Louis, MO USA; ^2^ Washington University in St. Louis School of Medicine, St. Louis, MO USA; ^3^ Washington University in St. Louis, St. Louis, MO USA

## Abstract

**Background:**

The use of biomarkers for early detection of Alzheimer disease (AD) is crucial for developing potential treatments. As neurons die, brain structures, including the hippocampus, shrink and the cerebrospinal fluid spaces ventricles expand. However, due to its small size, hippocampal volumes can be challenging to measure. The lateral ventricles are larger and easier to reproducibly segment, even in low resolution scans. In this study, we investigated whether baseline and longitudinal changes in lateral ventricle volume could predict baseline and longitudinal changes in hippocampal volume and in cognition, assessed by the clinical dementia rating (CDR®) sum of boxes.

**Method:**

We evaluated 518 participants enrolled in longitudinal studies at the Knight ADRC at Washington University in St. Louis (See Table 1). MRI and clinical data were obtained through OASIS (www.oasis‐brains.org). Lateral ventricular and hippocampal volumes were extracted and corrected for intracranial volume using FreeSurfer. Linear regression models were used to assess whether the baseline lateral ventricle volumes predict the baseline CDR sum of boxes or baseline hippocampal volume. A two‐step random coefficient model was used to assess whether the rate of change in lateral ventricle volume predicts the rate of change in the CDR sum of boxes or the rate of change in hippocampal volume.

**Result:**

We found that larger baseline lateral ventricle volumes predict smaller baseline hippocampal volumes (estimate β =‐0.04, p<0.0001, Table 2, Figure 1A) and worse baseline CDR sum of boxes (estimate β =0.000007, p=0.0024, Table 2, Figure 1C). Additionally, the rate of change in lateral ventricle volumes predicts the rate of change in hippocampal volume (estimates β =‐0.0221, p<0.0001, Table 2, Figure 1B) and rate of change in CDR sum of boxes (estimates β =0.0001, p<0.0001, Table 2, Figure 1D).

**Conclusion:**

Larger baseline lateral ventricles volumes predict smaller baseline hippocampal volumes and worse baseline CDR sum of boxes in an AD cohort. In addition, increased rates of enlargement of lateral ventricle volumes predict increased atrophy rates of the hippocampus and worsening CDR sum of boxes. Baseline and longitudinal lateral ventricle volumes could potentially be used to evaluate risk for AD in clinical populations.